# The Role of Quercetin as a Plant-Derived Bioactive Agent in Preventive Medicine and Treatment in Skin Disorders

**DOI:** 10.3390/molecules29133206

**Published:** 2024-07-05

**Authors:** Michał Kazimierz Zaborowski, Anna Długosz, Błażej Błaszak, Joanna Szulc, Kamil Leis

**Affiliations:** 1 Department of Food Industry Technology and Engineering, Faculty of Chemical Technology and Engineering, Bydgoszcz University of Science and Technology, 85-326 Bydgoszcz, Poland; 2 Faculty of Medicine, Collegium Medicum in Bydgoszcz, Nicolaus Copernicus University in Toruń, 87-100 Toruń, Poland

**Keywords:** quercetin, wound healing, skin treatment, antioxidant, anti-inflammatory, UV protection

## Abstract

Quercetin, a bioactive plant flavonoid, is an antioxidant, and as such it exhibits numerous beneficial properties including anti-inflammatory, antiallergic, antibacterial and antiviral activity. It occurs naturally in fruit and vegetables such as apples, blueberries, cranberries, lettuce, and is present in plant waste such as onion peel or grape pomace which constitute good sources of quercetin for technological or pharmaceutical purposes. The presented study focuses on the role of quercetin in prevention and treatment of dermatological diseases analyzing its effect at a molecular level, its signal transduction and metabolism. Presented aspects of quercetin potential for skin treatment include protection against aging and UV radiation, stimulation of wound healing, reduction in melanogenesis, and prevention of skin oxidation. The article discusses quercetin sources (plant waste products included), methods of its medical administration, and perspectives for its further use in dermatology and diet therapy.

## 1. Introduction

The directions of development in the food industry are strictly connected to the dominating trends in the food sector. Currently, worldwide tendencies point to sustainable development, with a focus on lab-grown food. It involves transferring production from the natural world to the laboratories in the face of growing ecological challenges like environmental degradation, climate change, and shrinking natural resources. The trend reflects the increased awareness and expectations of customers regarding sustainable development, and is a potential solution to the issue of raw materials recovery in the situation of the global raw material shortage and the growing problems with waste management [1]. The food industry fits perfectly into the latest developments with its new technologies for utilizing waste materials or enriching food with compounds obtained from waste [2,3,4]. That, in turn, results in further advancements like the health-promoting qualities of food, or the reduction and/or management of byproducts, particularly those from plant production, which are used as the main source of health-benefitting bioactive compounds like polyphenols [5].

**Figure 1:**
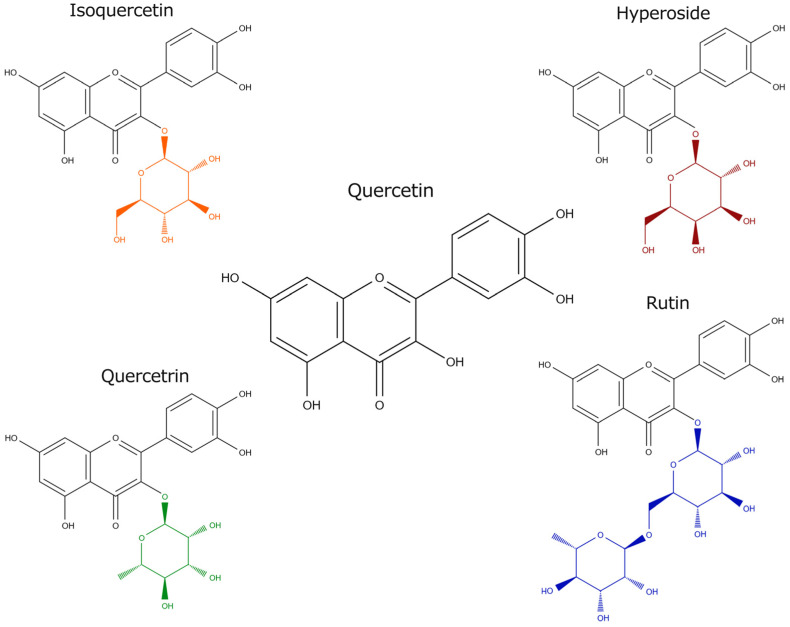
Structure of quercetin and its derivatives [11].

Quercetin (Que), 3,3′,4′,5,7-pentahydroxyflavone, is an organic compound in the group of flavonols which are a class of flavonoids. In nature, quercetin occurs as the aglycone of flavonoid glycosides forming isoquercetin with glucose, rutin with rutinose, hyperoside with galactose, and quercetrin with rhamnose (Figure 1).

Quercetin-3-O-glucoside, naturally occurring as a yellow plant pigment, is the most common Que glycoside [6]. The compound is sparingly soluble in water, and soluble in alcohol [7], lipids [8] and organic solvents [9]. Quercetin is not synthesized in the human body [10].

Quercetin is widely distributed in many plant-derived products; foods high in quercetin include, e.g., onion, capers, grapes or berries. Depending on the dietary habits, some of them can be the main sources of quercetin. However, the concentration of bioactive compounds, including quercetin, depends on numerous factors: plant maturity, harvest time, and farming techniques [12,13,14,15]. It should be noted that byproducts contain up to several times more quercetin than edible parts of plants and therefore, waste may be a good source of quercetin and other bioactive compounds used in food, chemical, beauty, and pharmaceutical industries. Table 1 presents the concentration of quercetin in foods and food industry byproducts.

**Figure 2:**
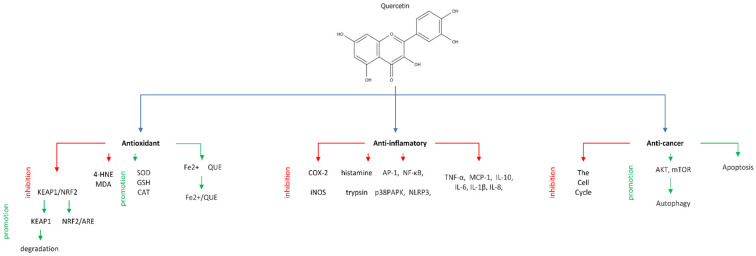
The biological activities of quercetin and its mechanism [37] (list of abbreviations used in Figure 2 and in the paper, with their descriptions, is in Table 2).

Quercetin is highly valued for the health benefits it offers (Figure 2). Its most important properties include the ability to neutralize free radicals, and its anticancer, anti-diabetic, anti-aging, antibacterial and anti-inflammatory effects observed even in cases of inflammation associated with chronic diseases [23,24,25,26,27,28,29,30,31,32]. Additionally, a positive impact on human skin has been confirmed in research [33,34,35,36]. Due to its beneficial health effects and the fact that it is easily available from plants and food industry byproducts, quercetin has the potential to be used in medicine, especially in disease prevention, treatment of skin diseases and injuries. Thus, the therapeutic potential of quercetin requires a summary providing an insight into the research conducted to date, especially into its impact on biochemical mechanisms and expression of enzymes and proteins, which is the main objective of this paper.

## 2. Anti-Aging Effects and UV Protection

Quercetin prevents degradation of collagen due to UV radiation in human skin and inhibits MMP-1 and COX-2 expression. Quercetin inhibits UV-induced AP-1 activity and NF-κB (Figure 2). Additionally, quercetin may reduce phosphorylation of ERK, JNK, and AKT, and STAT3Kinase assays using purified protein demonstrated quercetin’s ability to directly inhibit the activity of PKCδ and JAK2. This suggests its possible direct interaction with PKCδ and JAK2 in the skin, counteracting UV-induced aging. [35]. Research by Vicentini et al. [38] confirmed reduction in skin irritation due to UV radiation by inhibition of NF-κB and such inflammatory cytokines as IL-1β, IL-6, IL-8, and TNF-α. Kim et al. [39] point to the fact that quercetin in propolis reduces PDK-1 and AKT phosphorylation, which suggests its efficacy in preventing UV-induced photoaging. Furthermore, when combined with caffeic acid ester and apigenin, quercetin reduces PI3K activity, further enhancing its protective effect.

Findings from the clinical study conducted by Nebus et al. [40], in which oak quercetin formulated into an SPF 15 protective cream was used, demonstrated its protective effect on collagen and elastin with sustained proper degradation of waste proteins in aging skin. Patients in the study observed considerable improvement in skin parameters such as wrinkling, elasticity, smoothness, skin radiance and moisture.

The anti-aging effects of quercetin and its derivative, quercetin caprylate, were demonstrated in studies conducted by Chondrogianni et al. [41]. The substances showed the ability to activate the proteasome, which is responsible for the degradation of damaged proteins in human cells. Its activation is crucial for increasing resistance to oxidative stress and improving the viability of HFL-1 fibroblasts. Both compounds contributed to the rejuvenation of aging fibroblasts. which indicates their potential as anti-aging ingredients.

The research into using quercetin for UV protection started with studies of its function in plant tissues [42,43]. Choquenet et al. [43] described the increased level of photoprotection within the UVA range displayed by quercetin and its derivative (rutin) used in oil-in-water emulsion with 10% concentration. The effect was fortified when the flavonoids were used in association with titanium dioxide. Rajnochová-Svobodová et al. [44] demonstrated that quercetin and its derivative, taxifolin (dihydroquercetin), effectively reduce UVA-induced damage in skin fibroblasts and epidermal keratinocytes. These antioxidants prevent the formation of reactive oxygen species (ROS), depletion of GSH, activation of CASP3, and increase the expression of antioxidant proteins: HO-1, NQO1 and CAT. In a direct comparison, quercetin proved to be more effective than taxifolin, although at the highest concentration, it exhibited pro-oxidant properties. Additionally, Liu et al. [45] highlight the potential applications of dihydroquercetin in the treatment and prevention of skin diseases, including those caused by solar radiation, further emphasizing the importance of this substance in dermatology.

The protective effects against UVB radiation were the subject of studies by Vicentini et al. [38]. When quercetin was topically applied to the skin in the form of a water-in-oil microemulsion, it effectively penetrated the deeper layers of the epidermis without causing irritation. It significantly limited the depletion of glutathione induced by UVB exposure and reduced the activity of ultraviolet-induced metalloproteinase. The findings were subsequently confirmed in Casagrande et al. [46], where topically applied quercetin also mitigated glutathione depletion and reduced the activity of metalloproteinase and myeloperoxidase. Furthermore, research conducted by Zhu et al. [47] demonstrated that quercetin affected UVB-induced cytotoxicity in epidermal keratinocytes. The antioxidant blocked the production of reactive oxygen species (ROS) caused by radiation. By scavenging reactive oxygen species, quercetin protected the cell membrane and mitochondria, and slowed the leakage of cytochrome c, inhibiting keratinocyte apoptosis.

## 3. Anti-Melanogenesis and Skin Whitening

The primary mechanism responsible for pigmentation and skin color is melanogenesis; thus, halting, slowing and counteracting this process is fundamental for skin lightening and whitening efforts [48]. Based on in vivo and in vitro studies, Choi and Shin [36] did not demonstrate a whitening effect of quercetin in cosmetic products. However, quercetin plays a crucial role in blocking melanogenesis by inhibiting tyrosinase, a key enzyme that activates the pigmentation process [49,50,51]. Nonetheless, other studies indicate that quercetin can increase tyrosinase activity and promote melanogenesis [52,53,54], which may result from the concentration-dependent activity. Findings of the above studies seem to suggest that pure quercetin at concentrations higher than 20 μM reduces melanin concentration, while at concentrations of 10–20 μM, it increases melanin content. Melanin content also decreases at concentrations of 50–100 μM, but this fact seems to be associated with increased cellular toxicity, which can be reduced by using quercetin in combination with vitamin C and arbutin [36]. Additionally, the anti-melanogenic effect depends on the position of hydroxyl groups (-OH) in the compound structure and the position and type of sugar residues in quercetin derivatives [55,56,57,58].

Conversely, studies by Chondrogianni et al. [41] on quercetin and its derivative, quercetin caprylate, showed that both compounds induce changes in the physiological characteristics of cells, including a localized whitening effect.

## 4. Wound Healing, Reduction of Skin Irritation and Scarring Prevention

One of the earliest analyses regarding the impact of quercetin on reducing dermal wound healing time in rats was conducted by Gomathi et al. [59]. The authors divided the studied rats into a control group and a group treated with quercetin incorporated into collagenous matrix. The group subjected to quercetin treatment exhibited a greater potential to quench free radicals in the active inflammatory processes in skin wounds. Consequently, the study pointed out the quercetin potential as a wound healing-promoting agent when used in dressing material.

Gopalakrishnan et al. [60] emphasized the significant role of TGF-β1 and VEGF activation by quercetin in the wound healing acceleration process. Two groups of rats: a control group and a Que-treated group were subject to evaluation for the period of 14 days. The quercetin-treated group exhibited a faster rate of wound closure compared to the control group. In addition to activating the above-mentioned compounds, quercetin significantly attenuated TNF-α activity, supported fibroblast proliferation processes and collagen activity, and induced IL-10 levels. A similar study was conducted by Kant et al. [61] where 80 rats divided into a 4 groups (including a control group) were treated with dimethyl sulfoxide solutions and Que concentrations ranging from 0.03% to 0.3%. After 20 days, it was found that the administration of the polyphenol at the highest dose of 0.3% resulted in faster formation of granulation tissue, allowing for quicker wound closure by supporting angiogenic and proliferative processes within fibroblasts. Quercetin also contributed to the induction of, e.g., IL-10, VEGF, TGF-β1, simultaneously reducing TNF-α activity.

The mechanism of quercetin impact on the improvement in wound healing quality, was investigated by Doersch and Newell-Rogers [62]. Quercetin was found to exhibit the potential to reduce fibrosis without affecting the rate of healing. Quercetin reduced the level of integrin αV while increasing the expression of integrin β1. These changes in transmembrane receptor expression may affect processes such as cell proliferation and extracellular matrix production. Additionally, the presence of quercetin reduces the demand for extracellular matrix, thus contributing to easier wound healing and scar reduction.

Moravvej et al. [63] points out quercetin’s potential for scar treatments. Long et al. [64] showed that quercetin combined with X-ray radiation reduces collagen synthesis in fibroblasts, both healthy and scarred. Continuing their research on the properties of quercetin, Hosnuter et al. [65], demonstrated in a randomized controlled clinical trial the efficacy of onion extract containing quercetin in reducing scar hypertrophy, while not affecting itchiness. Further studies conducted by Ramakrishnan et al. [66] focused on the synergistic action of quercetin with vitamin D3 on isolated keloid fibroblasts. Quercetin was found to reduce cell proliferation and collagen synthesis while inducing apoptosis. In addition, Si et al. [67] demonstrated that quercetin can suppress keloid resistance to radiotherapy by inhibiting the expression of the HIF-1 and interacting with the PI3K/AKT pathway, reducing AKT phosphorylation.

Yin et al. [68] demonstrated the effectiveness of quercetin in the process of pressure ulcer healing. Their in vitro and in vivo analyses of induced pressure ulcers in mice provided evidence as to quercetin participation in stimulating processes responsible for wound edge closure by inhibiting interference with the MAPK pathway. Additionally, quercetin was found to reduce cellular infiltration and decrease the concentration of inflammatory cytokines.

As suggested by Karuppagounder et al. [69], Beken et al. [70], and Hou et al. [71], the anti-inflammatory and antioxidant properties of quercetin can be utilized in the treatment of atopic dermatitis (AD). The course and development of AD are associated with the action of epithelial-derived cytokines [72]. In studies conducted by Beken et al. [70], the use of quercetin in prepared keratinocytes resulted in a decrease in the expression of cytokines such as IL-1β, IL-6, IL-8, and TSLP, and a simultaneous increase in the expression of SOD1, SOD2, GPX, CAT, and IL-10. An increase in mRNA expression of E-cadherin and occludin was also observed, along with a decrease in the expression of matrix metalloproteinases: MMP-1, MMP-2, and MMP-9. Additionally, inhibition of ERK1/2 phosphorylation, and MAPK was noted, as well as decreased expression of nuclear transcription factor NF-κB, with no effect on STAT6. The mechanism of action of quercetin had been previously determined by Cheng et al. [73], who focused on its impact on retinal pigment epithelial cells. Hou et al. [71] demonstrated that quercetin effectively lowers expression levels of cytokines such as CCL17, CCL22, IL-4, IL-6, IFN-γ, and TNF-α.

Quercetin demonstrates the ability to inhibit pro-inflammatory cytokines induced by *Propionibacterium acnes* bacteria. Lim et al. [33] showed that it suppresses TLR-2 production and inhibits phosphorylation of p38, ERK, and JNK MAPK kinases. Additionally, a decrease in mRNA levels for MMP-9 was observed. In vivo studies showed quercetin-induced reduction in thickness of erythema and swelling.

Liu et al. [74] developed quercetin-loaded liposomes in gels (QU-LG) to investigate their possible therapeutic effect on cutaneous eczema due to favorable antioxidant activity and anti-inflammatory effects of quercetin. Que was encapsulated in liposomes and evenly dispersed in sodium carboxymethyl cellulose hydrogels in order to enhance its bioavailability and efficiency of its dermal delivery. The research demonstrated that quercetin-containing liposomes-in-gel (QU-LG) applied to the skin of mice suffering from skin eczema exhibited good stability and adhesion to the skin. In the antioxidant test, QU-LG inhibited the production of malondialdehyde (MDA) in the liver better than the commercially available drug, dexamethasone acetate cream. Compared to untreated mice, mice treated with QU-LG showed a statistically significant reduction in dermatopathological symptoms. The results suggest that QU-LG exhibits good antioxidant activity in vivo and in vitro and that it can be used in the prevention and treatment of cutaneous eczema.

Maramaldi et al. [34] and Kurek-Górecka [75] investigated the use of phytosomes, which are alternatives to liposomes, as formulations enhancing the bioavailability of active ingredients. Quercetin in the form of phospholipids significantly reduced erythema and wheal diameter [34]. The study of Lu et al. [76] into quercetin-loaded niosomes described improved solubility, photostability, and skin penetration ability compared to conventional methods of delivering active ingredients. Li et al. [77] developed a xerogel utilizing polyvinyl alcohol (PVA) and quercetin-borate nanoparticles as the crosslinking agent. The produced xerogel films exhibited high bacteriostatic properties, high antioxidant potential, and accelerated skin regeneration.

Nalini et al. [78] compared the effects of quercetin and quercetin-loaded chitosan nanoparticles on the healing processes of open wounds. An accelerated healing process was observed in the studied rodents, due to inhibition of inflammatory cytokines, promotion of angiogenesis, and inhibition of free radicals. Increased levels of hydroxyproline and hexalin indicated enhanced reepithelialization. Quercetin used in monotherapy showed lower effectiveness than the mixture of quercetin-loaded alginate. Chitosan nanoparticles with a concentration of 0.075% showed the highest efficacy.

Yang et al. [79] investigated the association between quercetin and histamine, a compound that triggers inflammation. The study demonstrated a direct interaction between histamine H4 receptors and quercetin. It was found that quercetin inhibits IL-8 mRNA expression in keratinocyte and the scratching behavior-induced compound 48/80. Additionally, quercetin reduces calcium influx (Ca^2+^) induced by the H4 receptor through the TRPV1 channel, which limits itching, inflammation, and discomfort sensation.

Studies conducted by Katsarou et al. [80] did not show a beneficial effect of quercetin on sodium-lauryl-sulfate-induced skin irritation. It was observed that quercetin did not restore the protective barrier function of the skin, normalize transepidermal water loss, nor reduce erythema to levels observed before irritation.

Due to the more challenging wound healing process in cases of diabetes, studies have investigated the wound-healing potential of quercetin in diabetic rats [81,82]. Fu et al. [81] administered quercetin-containing medication to diabetic rats at various concentrations and observed reduced levels of inflammatory cytokines and the number of iNOS-positive cells, as well as increased activity of CD206-positive cells and intensified angiogenesis processes. The researchers attributed these effects to the influence of quercetin on inducing a shift in macrophage phenotype towards M2. Kant et al. [82] also evaluated the effectiveness of quercetin on wounds in diabetic rats. The applied quercetin (0.3%) ointment accelerated wound healing time and induced, e.g., VEGF and TGF-β, at the same time reducing levels of MMP-9 and TNF-α. The researchers also observed higher levels of GAP-43 resulting from polyphenol application.

## 5. Protection against Skin Oxidation, Anti-Inflammatory and Antiseptic Properties

Numerous studies have described not only the anti-inflammatory and antioxidant effect of quercetin on skin but also its general antioxidant activity [32,83,84]. Experimental studies by Tang et al. [85] confirmed the effectiveness of quercetin in inhibiting TNF-α, IL-1β, and IL-6 cytokines. The theoretical calculations illustrated that the oxygen atom on B rings may be the main site of electron cloud density changes, which results in ROS scavenging effects of quercetin. Ha et al. [86] demonstrated, that quercetin 3-O-β-D-glucuronide possesses protective effects on skin, including anti-inflammatory and antioxidant actions against UVB- or H_2_O_2_-induced oxidative stress. It reduces the expression of pro-inflammatory genes (COX-2, TNF-α) in stressed HaCaT cells as well as increasing Nrf2 expression and inhibiting melanin production in α-MSH-treated B16F10 cells.

Quercetin, combined with rutin and curcumin, loaded on porous copper oxide nanorods, exhibits not only anti-inflammatory properties but also bacteriostatic and bactericidal properties. Mansi et al. [87] demonstrated Que nanocomposite’s effective antagonism against bacteria such as *Staphylococcus aureus*, *Bacillus subtilis*, *Salmonella typhi*, *Pseudomonas aeruginosa*, *Escherichia coli*, and *Klebsiella pneumoniae*. The inhibitory action against *Pseudomonas aeruginosa* and *Staphylococcus aureus* was confirmed by Chittasupho et al. [88]. Ramzan et al. [89] utilized nanoparticles composed of quercetin and its copper complex, employing polycaprolactone (PCL) as a structural material, for treating skin infections. Studies showed that the nanoparticles have strong bactericidal effect against *Staphylococcus aureus* and stimulate epidermal regeneration without causing skin irritation. This points to the potential use of quercetin as an active agent in the treatment of impetigo and as an alternative to widely used ciprofloxacin. Quercetin delivered in the form of oil-based nanostructured lipid carriers also showed efficacy against *Staphylococcus aureus* [90]. Lúcio et al. [91] described the synergistic action of quercetin with omega-3 fatty acids, where bioactives in the form of nanostructured lipid carriers and hydrogels were exhibited with high stability and skin permeability.

Quercetin, combined with other antioxidants, occurring in the extract of sumac (*Rhus coriaria*), shows strong antibacterial activity. Gabr and Alghadir [92] demonstrated the antibacterial effect of the extract against *Staphylococcus aureus* and *Pseudomonas aeruginosa*. Additionally, the extract reduced inflammation, regulated the activity of MMP-8 and MPO enzymes, supported wound contraction, and promoted collagen and hydroxyproline deposition. Extracts isolated from *Syncarpia hillii* leaves, containing the quercetin glycoside (quercitrin), also exhibited high antibacterial activity against both Gram-positive and Gram-negative bacteria, including staphylococci and *Enterococcus faecalis*. The leaves are used in traditional herbal medicine for treating wounds and skin infections [93]. Extracts rich in quercetin and its glycosides from the *Opuntia genus* (*Opuntia* Spp.) also show antibacterial properties in wound treatment [94], as do extracts from *Bridelia ferruginea* [95] and *Spermacoce princeae*, which additionally exhibit UV-protective effects [96].

Quercetin exhibits antibiofilm effectiveness. Biofilms are bacterial aggregations that can grow on different surfaces. Biofilms colonize wounds, protect the pathogen from host defenses and obstruct antibiotic delivery, thereby weakening wound healing [97]. Mu et al. [98] claim that quercetin and other secondary metabolites isolated from plants have demonstrated varying levels of biofilm inhibition in Gram-negative pathogens. In their studies [98,99] quercetin and extracts rich in this bioactive compound exhibited a concentration-dependent reduction in *Staphylococcus epidermidis* biofilm formation. Quercetin reduced biofilm formation up to 95.3% at the concentration of 500 μg mL^−1^. Gopu et al. [100] tested quercetin in case of biofilm formation of different pathogens, responsible for food spoilage. Their studies, conducted for quercetin at different concentration (20–80 μg mL^−1^), showed 13–72%, 8–80%, and 10–61% reduction in biofilm biomass of *K. pneumoniae*, *P. aeruginosa*, and *Y. enterocolitica,* respectively. In their studies, Musini et al. [97] highlighted the antibiofilm activity of quercetin against the drug-resistant pathogen *S. aureus*. Since *S. aureus* is an important virulence factor influencing its persistence in both the environment and the host organism and is responsible for biofilm-associated infections, the antibiofilm activity of quercetin is a promising solution for antibiotic-resistant strains of this bacteria.

## 6. Bioavailability, Safety of Use, and Potential Harmful Effects

The effectiveness of quercetin and its derivatives in treating skin diseases may be compromised by its absorbability [101]. Studies conducted by Hung et al. have demonstrated that quercetin is better absorbed through photoaged skin, likely because UV exposure disrupts the barrier functions of the skin [102]. Lin et al. [103] compared the transdermal absorption levels of quercetin and its derivatives, including polymethoxylated quercetin (QM). It was shown that the structure of the compound has a crucial impact on transdermal absorption. Derivatives with higher lipophilicity more easily penetrated the skin barrier. Furthermore, the sugar moiety of glycosides significantly affects skin permeability, with those having -OH groups potentially forming hydrogen bonds with ceramides in the epidermis. QM demonstrated the highest level of permeation, suggesting it as the best delivery form of quercetin for topical applications.

Quercetin is considered safe for daily consumption by food safety authorities, including the U.S. FDA—Food & Drug Administration [104]. The average daily intake of quercetin from food is approximately 20–40 mg. The daily dosage of quercetin in dietary supplements ranges from 50 to 500 mg. Detection of this compound in plasma is possible about 15–30 min after consuming a 250 mg or 500 mg chewable quercetin preparation. The maximum concentration is reached after 120–180 min (levels return to baseline after 24 h) [105]. In studies, doses of 500–1000 mg of quercetin are typically used. It has been proven that such an amount of quercetin, up to 1000 mg, taken over several months does not adversely affect blood parameters, liver and kidney function, or serum electrolyte levels [106].

In addition to many health benefits, potential health risks associated with the use of quercetin have also been noted [107,108]. Studies have shown a negative impact of quercetin supplementation for neurodegenerative prevention, aimed at protecting nerve cells. It has been demonstrated that high exposure to quercetin can lead to a reduction in intracellular glutathione levels, as well as changes in the genes responsible for intracellular processes [107]. Different studies suggest potential cytotoxic activity of quercetin, induced by inhibiting the action of specific genes in the presence of gamma radiation doses [108]. In conclusion, it is important to emphasize that the risks associated with quercetin use are minimal compared to its benefits. Long-term consumption of quercetin is not recommended, however, for individuals prone to hypotension (low blood pressure) and those with impaired blood clotting [109].

## 7. Summary

Quercetin exhibits many health-promoting, antioxidant, and therapeutic properties (Table 3). While research confirms the potential use of its various forms (e.g., extract, emulsion, aqueous extracts) in skin therapy, the variety of methods for obtaining quercetin even broadens its possible application in medicine. The cited studies suggest that research on the use of natural sources of quercetin in treatment of skin diseases, specifically reducing oxidation processes, aging, melanogenesis, scarring, accelerating wound healing, and protection against UV radiation, is well founded. Advances in the research will undoubtedly contribute to the development of new dermatological preparations and therapies. Among many studies in the field of development new materials, many of them mention incorporating quercetin or extracts containing this compound into wound healing and treatment of skin diseases [110,111,112]. This direction seems to be the closest to commercialization and therefore the appearance of products (hydrogels, patches, wound dressings etc.) should take place in the near future. Obtaining quercetin from waste from the food industry and constantly improving technologies and extraction techniques will allow for greater availability of this compound, not only in medicine, but also in pharmaceuticals and the food sector.

## Figures and Tables

**Table 1:** Chosen quercetin sources.

Reference	Amount [mg 100 g^−1^]	Form	Product
[16]	4.0 *	Fresh crop	Apple
[16,17]	14.0–23.6 *	Fresh crop	Asparagus
[16,18]	2.4–14.6 *	Fresh crop	Blueberry
[18]	15.8 *	Fresh crop	Whortleberry
[18]	14.6 *	Fresh crop	Lingonberry
[18]	8.9 *	Fresh crop	Chokeberry
[18]	6.3 *	Fresh crop	Rowanberry
[18]	5.6 *	Fresh crop	Crowberry
[18]	0.6 ***	Juice	Elderberry
[16,18]	12.1–25.0 *	Fresh crop	Cranberry
[16]	17.4 *	Fresh crop	Cherry
[19,20]	0.46 ***	Juice from fresh Italian grape (Chianti)	Grape
[21]	54–119 **	Grape waste (pomace): Methanolic extract from waste (skins, stalks, seeds)	
[20]	0.62–1.02 **	Dried grape leaves	
[20]	0.68–0.82 **	Dried grape stems	
[16]	3.16 ***	Beverage	Red wine
[16]	22.6 *	Fresh crop	Kale
[16]	14.7 *	Fresh crop	Lettuce
[17]	12.0 *	Fresh crop	Romaine lettuce
[17]	30.6–10.3 *	Fresh crop	Red leaf lettuce
[17]	1.6 *	Fresh crop	Tomato
[16,17]	11.0–45.0 *	Fresh crop	Yellow onion
[22]	792.0 **	Waste (peels): water and ethanol extracts	
[22]	41.9 **	Edible part water and ethanol extracts	Red onion
[22]	1625.0 *	Waste (peels) water and ethanol extracts	
[22]	40.0 **	Edible part water and ethanol extracts	Red shallot
[22]	1161.0 *	Waste (peels) water and ethanol extracts	

* Amount presented in fresh weight (FW). ** Amount presented in dry weight (DW). *** Amount presented in mg/100 mL.

**Table 2:** List of abbreviations and their descriptions.

Full Name	Abbreviation
Kelch-like ECH-associated protein 1	KEAP1
nuclear respiratory factor 2	NRF2
antioxidant response element	ARE
4-hydroxynonenal	4-HNE
3,4-methylenedioxyamphetamine	MDA
superoxide dismutase	SOD
Glutathione	GSH
Catalase	CAT
Quercetin	QUE
cyclooxygenase-2	COX-2
inducible nitric oxide synthase	INOS
activator protein-1	AP-1
p38 mitogen-activated protein kinases	p38PAPK
nuclear factor-κB	NF-κB
NLR family pyrin domain containing 3	NLRP3
tumor necrosis factor-α	TNF-α
monocyte chemoattractant protein-1	MCP-1
interleukin-10	IL-10
interleukin-6	IL-6
interleukin-1β	IL-1β
interleukin-8	IL-8
protein kinase B	AKT
mammalian target of rapamycin	mTOR
extracellular signal-regulated kinase family	ERK
extracellular signal-related kinases 1 and 2	ERK1/2
kinase c-Jun N terminal	JNK
signal transducers and activator of transcription 3	STAT3
protein kinase Cδ	PKCδ
Janus kinase-2	JAK-2
phosphoinositide-dependent protein kinase-1	PDK-1
heme oxygenase-1	HO-1
human fetal lung fibroblast-1	HFL-1
transforming growth factor-β1	TGB-β1
vascular endothelial growth factor	VEGF
transforming growth factor-β1	TGF-β1
hypoxia-inducible factor-1	HIF-1
phosphoinositide 3-kinase	PI3K
mitogen-activated protein kinases	MAPK
matrix metalloproteinase-1	MMP-1
matrix metalloproteinase-2	MMP-2
matrix metalloproteinase-9	MMP-9
extracellular signal-regulated kinase 1	ERK1
signal transducers and activator of transcription 6	STAT6
CC chemokine ligand 17	CCL17
CC chemokine ligand 22	CCL22
interleukin-4	IL-4
interferon gamma	IFN-γ
Toll-like receptor-2	TLR-2
growth-associated protein-43	GAP-43
matrix metalloproteinase-8	MMP-8
Myeloperoxidase	MPO
caspase 3	CASP3
NAD(P)H quinone dehydrogenase 1	NQO1
Thymic stromal lymphopoietin	TSLP
Superoxide dismutase 1	SOD1
Superoxide dismutase 2	SOD2
glutathione peroxidase	GPX
transient receptor potential cation channel subfamily V member 1	TRPV1

**Table 3:** Mechanism of action of quercetin.

Reference	MOA	Compound
Anti-aging effects and UV protection		
[35]	Inhibition of MMP-1 and COX-2 expression	quercetin
[35,38]	Inhibition of AP-1 and NF-κB activity	quercetin
[38]	Inhibition of IL-1β, IL-6, IL-8, and TNF-α inflammatory cytokines	quercetin
[39]	Inhibition of PDK-1 and AKT phosphorylation	quercetin, caffeic acid ester and apigenin
[43]	Reduction of UVA radiation effects	quercetin + rutin + titanium dioxide
[44]	Prevention of ROS creation, GSH depletion, CASP3 activation. Increased HO-1, NQO1, CAT expression	quercetin
[38,46]	Reduction in GSH depletion, reduction in metalloproteinase activity	quercetin
[47]	Blocked ROS production, slowed leakage of cytochrome c, inhibition of keratinocyte apoptosis	quercetin
[41]	Activation of proteasome, improvement in HFL-1 fibroblasts viability	quercetin, quercetin caprylate
Anti-melanogenesis and skin-whitening		
[49,50,51]	Inhibition of tyrosinase	quercetin
[36]	Reduction in melanin content	quercetin + vitamin C + arbutin
[52,53,54]	Upregulation of tyrosinase activity	quercetin
Wound healing, reduction in skin irritation and scarring prevention		
[59]	Inhibition of free radicals’ activity	quercetin
[60]	TGB-β1 and VEGF activation, Promotion of fibroblast proliferation, TNF-α attenuation, inducing IL-10 levels	quercetin
[61]	Increasing IL-10, VEGF, TGF-1 expressions, and decreasing expressions of TNF-α	quercetin in DMSO
[62]	Reduction in integrin αV level; increasing integrin β1 expressions	quercetin
[66]	Reduction in cell proliferation and collagen synthesis; inducing apoptosis	quercetin + vitamin D
[64]	Inhibition of collagen synthesis in fibroblasts	quercetin + X-ray
[67]	Inhibition of HIF-1 expression Reduction in AKT phosphorylation	quercetin
[65]	Improvement in hypertrophic and keloid scars	quercetin
[68]	Inhibition of MAPK signaling pathway; Reduction in inflammatory cytokines concentration	quercetin
[70,73]	Reduction in IL-1β, IL-6, IL-8 expressions Increasing SOD1, SOD2, GPx, catalase expressions, IL-10 Increasing mRNA, E-cadherin, occludin expressions Decreasing MMP1, MMP2 and MMP9 expressions Inhibition of ERK1/2 MAPK phosphorylation Decreasing NF-κB expression	quercetin
[71]	Reduction in CCL17, CCL22, IL-4, IL-6, IFN-γ and TNF-α expressions	quercetin
[33]	Reduction in TLR-2 production Inhibition of p38, ERK and JNK MAPK Decreasing mRNA level for MMP-9	quercetin
[74]	Inhibition of malondialdehyde production	quercetin encapsulated in liposomes
[78]	Inhibition of inflammatory cytokines, enhancing free radical scavenging ability, increasing reepithelialization	quercetin, chitosan, alginate
[79]	Decreasing histamine 4 receptor-induced calcium influx through the TRPV1 channel	quercetin
[81]	Decreasing inflammatory cytokines levels and INOS-positive cells Increasing CD206-positive cells activity Induction of macrophage polarization to M2	quercetin
[82]	Induction of VEGF, TGF-β Increasing GAP-43 concentration Reduction in MMP-9 and TNF-α	quercetin
Protection against skin oxidation, anti-inflammatory, and antiseptic properties		
[85]	Changing electron cloud density, Inhibition of TNF-α, IL-1β and IL-6 cytokines	quercetin
[87]	Antimicrobial activity	quercetin on CuO nanorods
[89]	Antimicrobial activity	quercetin + copper complex + polycaprolactone
[90]	Antimicrobial activity	quercetin-loaded lipid carriers
[92]	Antimicrobial activity regulation of MMP-8 and MPO activity Increasing collagen and hydroxyproline deposition	extract of *Rhus coriaria* (quercetin)
[93]	Antimicrobial activity	Extract of *Syncarpia hillii* (Querciturone)
[94]	Antimicrobial activity	Extract of *Opuntia* spp. (quercetin and its glycosides)
[95]	Antimicrobial activity	Extract of *Bridelia ferruginea* (quercetin)
[96]	Antimicrobial activity UV protection	Extract of z *Spermacoce princeae* (quercetin)

## Data Availability

No new data were created.
